# Influence of third-body particles originating from bone void fillers on the wear of ultra-high-molecular-weight polyethylene

**DOI:** 10.1177/0954411916651461

**Published:** 2016-06-16

**Authors:** Raelene M Cowie, Silvia Carbone, Sean Aiken, John J Cooper, Louise M Jennings

**Affiliations:** 1 Institute of Medical and Biological Engineering, School of Mechanical Engineering, University of Leeds, Leeds, UK; 2 Biocomposites Ltd., Keele, UK

**Keywords:** Knee replacement, third-body damage, calcium sulfate, bone void filler, wear, ultra-high-molecular-weight polyethylene, in vitro

## Abstract

Calcium sulfate bone void fillers are increasingly being used for dead space management in infected arthroplasty revision surgery. The presence of these materials as loose beads close to the bearing surfaces of joint replacements gives the potential for them to enter the joint becoming trapped between the articulating surfaces; the resulting damage to cobalt chrome counterfaces and the subsequent wear of ultra-high-molecular-weight polyethylene is unknown. In this study, third-body damage to cobalt chrome counterfaces was simulated using particles of the calcium sulfate bone void fillers Stimulan^®^ (Biocomposites Ltd., Keele, UK) and Osteoset^®^ (Wright Medical Technology, TN, USA) using a bespoke rig. Scratches on the cobalt chrome plates were quantified in terms of their density and mean lip height, and the damage caused by the bone void fillers was compared to that caused by particles of SmartSet GMV PMMA bone cement (DePuy Synthes, IN, USA). The surface damage from Stimulan^®^ was below the resolution of the analysis technique used; SmartSet GMV caused 0.19 scratches/mm with a mean lip height of 0.03 µm; Osteoset^®^ led to a significantly higher number (1.62 scratches/mm) of scratches with a higher mean lip height (0.04 µm). Wear tests of ultra-high-molecular-weight polyethylene were carried out in a six-station multi-axial pin on plate reciprocating rig against the damaged plates and compared to negative (highly polished) and positive control plates damaged with a diamond stylus (2 µm lip height). The wear of ultra-high-molecular-weight polyethylene was shown to be similar against the negative control plates and those damaged with third-body particles; there was a significantly higher (*p* < 0.001) rate of ultra-high-molecular-weight polyethylene wear against the positive control plates. This study showed that bone void fillers of similar composition can cause varying damage to cobalt chrome counterfaces. However, the lip heights of the scratches were not of sufficient magnitude to increase the wear of ultra-high-molecular-weight polyethylene above that of the negative controls.

## Introduction

Over 140,000 primary arthroplasty procedures are carried out in the National Health Service (NHS) annually^1^ with the aim of reducing pain and restoring joint function in patients with osteoarthritis. Despite an estimated survivorship of greater than 95% at 10 years, failure of metal-on-polyethylene implants most commonly occurs as a result of wear of the ultra-high-molecular-weight polyethylene (UHMWPE) component^2^ leading to aseptic loosening. To reduce the potential for aseptic loosening due to wear debris–induced osteolysis, the wear of the UHMWPE component should be minimised. Counterface roughness is one of the most important determinants of wear volumes and the number and morphology of micron and sub-micron size particles generated.^3,4^ It only requires a single hard particle to enter the contact surfaces to cause a single scratch of 2 µm depth to produce a large increase in UHMWPE wear^3^ with the wear rate primarily dependent on the scratch lip height.^4^ Damage to the cobalt chrome counterface causes a change in the dominant wear mechanism from adhesive to abrasive, which accelerates UHMWPE wear.^5^ Clinically, femoral counterfaces can be roughened by bone cement particles, bone particles, and metallic debris, which when trapped between the articulating surfaces can cause damage to the bearing surfaces and accelerate wear.^6^ The presence of third-body particles such as bone cement, bone fragments, or porous-coating beads in retrieved devices and surrounding tissue^7^ has been widely reported with the particles originating during device implantation, from the device itself, or from failure of the cement mantle.^7,8^

There have been several approaches to experimentally recreating the damage to articulating surfaces by third-body particles in total joint replacements and subsequently determining the wear of UHMWPE. In order to generate reproducible scratches which replicate those observed on clinical retrievals, the most reliable method has been to use a diamond stylus to directly scratch the counterface surface.^3,5,6,9,10^ Other approaches have included tumbling the implant in a material such as alumina powder^11,12^ or roughening with silicone carbide paper.^13–16^ However, these approaches cannot be controlled as effectively as scratching directly with a diamond stylus. Several studies have introduced third-body particles into the lubricant used for wear testing.^17^ These particles have been derived from bone^18–20^ or synthetic materials such as bone cement^8,21^ or aluminum oxide particles.^22,23^ However, when dosing the lubricant, it is very difficult to control which, if any, of the particles become entrained into the articulating surfaces. The challenge is therefore to recreate clinically relevant surface damage using third-body particles, to quantify this damage using appropriate surface topographical measurements, and to determine the wear of UHMWPE.

The third-body damage method used in this study was adapted from previous work by Minakawa.^24^ In Minakawa’s study, particles of bone and bone cement were trapped between a truncated cone UHMWPE pin and flat plates composed of different materials typically used as the hard bearing material in joint arthroplasty. The pin was axially loaded using a materials testing machine, and the plate was pulled beneath the pin via a pulley system recreating third-body damage. To give the surface damage clinical relevance, the bearing materials and third-body particles were similar to those used in joint arthroplasty and the third-body particles were controlled in a size range found in retrieved implants.

In this study, however, the third-body particles used were derived from calcium sulfate bone void fillers (BVFs), which are increasingly being used in revision procedures for the management of infected prostheses.^25^ Superficial infection of total knee replacements can often be successfully treated using systemic antibiotics while deep infection is more usually treated with debridement and washout often resulting in revision of the implant.^26–29^ Such debridement can lead to significant dead space which becomes a potential source of re-infection. To manage this dead space, a variety of materials have been considered including polymethyl methacrylate (PMMA) bone cement and resorbable materials such as hydroxyapatite, collagen, fibrin, various polymers such as polylactides, and calcium sulfate.^30,31^ Calcium sulfate BVFs provide a low-cost, synthetic alternative to allogenic bone grafts; they are biocompatible, resorbable, and osteoconductive and do not elicit a foreign body or inflammatory response.^32^ They resorb in approximately 6–8 weeks when implanted in a bone void^33,34^ and may be loaded with antibiotics which are slowly and locally released as the calcium sulfate resorbs.^35,36^ However, there are concerns over the potential for loose beads of BVFs to migrate into the joint space causing third-body damage and accelerating UHMWPE wear. For one material to scratch another, the hardness of the scratching material must be similar to or greater than that of the scratched material. Calcium sulfate has a lower hardness compared to materials already known to damage metal counterfaces, including barium sulfate and zirconium dioxide, which are commonly added to PMMA bone cement to improve the radiopacity;^8,18^ however, depending on the processing route, calcium sulfate BVFs can contain impurities.^37^

The aim of this study was to determine the surface damage to cobalt chrome counterfaces if third-body particles originating from BVFs or PMMA bone cement were to become trapped between the articulating surfaces of a joint replacement. Following damage simulation, the wear performance of UHMWPE was assessed against the cobalt chrome counterfaces using a multi-axial pin on plate reciprocating rig with tribological and kinematic conditions to reflect the contact pressure and cross-shear in total knee replacements.^38^ Due to the lower hardness of calcium sulfate compared with materials already used in joint replacement procedures such as the barium sulfate and zirconium dioxide in PMMA bone cement, it was hypothesised that if pellets of BVFs were to become trapped between the articulating surfaces of a total knee replacement there would be no influence on the wear of the UHMWPE.

## Materials and methods

In accordance with the previous methodology described by Minakawa,^24^ the study was split into two phases: the first phase comprised third-body damage to cobalt chrome counterfaces with different third-body particles; the second phase consisted of experimental wear simulation against the damaged surfaces. This approach gave greater control over the number of particles trapped between the articulating surfaces^18^ and is accepted practice to recreate damage and subsequently perform wear testing.^3,5,10^

### Materials

The components and third-body materials used are shown in Tables 1 and 2.

**Table 1.** Components used in this study.

Components
GUR 1020 UHMWPE pins 3 mm flat contact face (conventional, unsterilised)
GUR 1020 UHMWPE pins 5 mm flat contact face (conventional, unsterilised)
Cobalt chrome plates (*R_a_* < 0.01 µm)

UHMWPE: ultra-high-molecular-weight polyethylene.

**Table 2.** Third-body particles used for damage simulation.

Third-body particles
PGCS: Stimulan^®^ bone void filler beads (3 mm), Biocomposites Ltd., Keele, UK
MGCS: Osteoset^®^ bone void filler pellets (3 mm), Wright Medical Technology, TN, USA
PMMA: SmartSet GMV^®^ Gentamicin bone cement (+10% barium sulfate), DePuy Synthes, IN, USA, prepared to 500–1000 µm particles

PGCS: pharmaceutical grade calcium sulfate; MGCS: medical grade calcium sulfate; PMMA: polymethyl methacrylate.

Third-body particles were generated from polymerised PMMA-GMV (SmartSet GMV PMMA bone cement, DePuy Synthes Joint Reconstruction, IN, USA) with 10% barium sulfate, cement particles were filtered and those in the size range of 500–1000 µm diameter were used for damage simulation; Stimulan^®^ a recrystallised pharmaceutical grade of calcium sulfate—PGCS (Biocomposites Ltd, Keele, UK) and Osteoset^®^ a medical grade calcium sulfate—MGCS (Wright Medical Technology, TN, USA). The brittle BVFs were crushed prior to testing.

**Figure 1.**
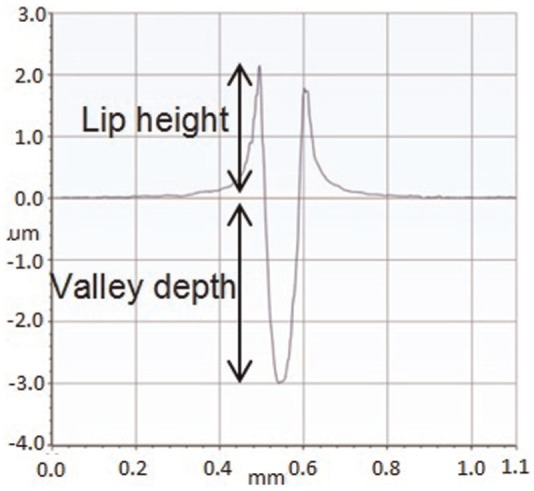
Positive control scratches damaged with a diamond stylus (mean lip height 2 µm), measurement taken with a Bruker NPFlex White Light Interferometer.

The pins used in the damage simulation and wear testing reflected the grade of polyethylene used in total knee replacement, GUR 1020 UHMWPE^38^ (conventional, unsterilised) and had a truncated cone geometry. Those used for damage simulation had a flat 3 mm diameter contact face and those used for wear simulation had a flat 5 mm diameter contact face. Pins used for wear simulation were soaked in sterile water for a minimum of 2 weeks prior to testing to maximise their water uptake. Highly polished (mean surface roughness, *R_a_* < 0.01 µm) cobalt chrome (CoCr) alloy plates were used as negative control counterfaces and for damage simulation. Discrete scratches were created on the positive control CoCr plates using a diamond stylus with a spherical tip radius of 200 µm. The scratches on the positive controls (Figure 1) had a spacing of 1.5 mm and an average lip height of 2 µm which is comparable in magnitude to scratches seen on explanted femoral heads^9^ and consistent with previous studies.^10^

### Methods

#### Phase 1: third-body damage simulation

**Figure 2.**
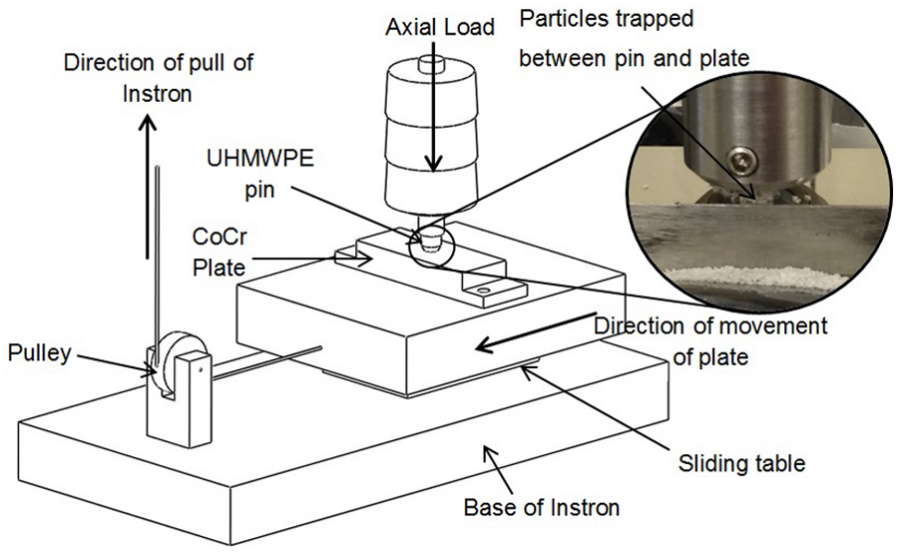
Schematic of the damage simulation rig with third-body particles trapped between UHMWPE pin and cobalt chrome plate (inset).

The protocol used was adapted from and validated against previous work by Minakawa.^24^ Using a bespoke rig (Figure 2), third-body particles were trapped (in excess) between an UHMWPE pin and a highly polished CoCr plate. A load of 120 N was applied axially through the pin to trap the particles between the pin and plate and then using an Instron 3365 Materials testing machine (Instron, MA, USA), the plate was pulled beneath the pin at 8 mm/min to damage the surfaces. To create a worst case test, damage simulation was carried out without lubricant.

In each region of damage, the particles were passed over the plate five times in the same direction and five discrete regions of damage were generated. Following damage simulation, the surface topography of the plates was analyzed using both two-dimensional (2D) contacting profilometry and white light interferometry. The surface roughness was determined using a PGI 800 contacting Form Talysurf profilometer (Taylor Hobson, Leicester, UK) with a 2 µm conical tip stylus; the instrument had a *z* resolution of 3.2 nm and a *z* repeatability of 0.12 nm. Least squares line form fitting was used to remove the background waviness of the surface and where appropriate filtering was used in line with ISO 4288:1998. The surface roughness parameters of interest were the mean surface roughness (*R_a_*), the maximum profile height above the mean line averaged over appropriate sampling lengths (*R_p_*), and the maximum valley depth (*R_v_*). These parameters were chosen to give a measure of the overall surface damage caused by the different third-body particles as previous studies have demonstrated an exponential relationship between increasing *R_p_* of scratches on a metal counterface and the wear of UHMWPE against the damaged surfaces.^6^ The mean lip height of the scratches and the density of scratches were determined from measurements taken by sampling the surface using a non-contacting Bruker NPFlex optical profiler (Bruker, MA, USA) with a 10× lens which had an optical resolution of 0.9 µm and a vertical resolution of <0.15 nm.^39^ Images of the surface damage on the plates were captured at 63× magnification using a PixeLINK camera in combination with a Nikon SMZ800 stereomicroscope with illumination of the samples via an external light source. Four replicates were completed for each third-body material.

#### Phase 2: experimental wear simulation

**Figure 3.**
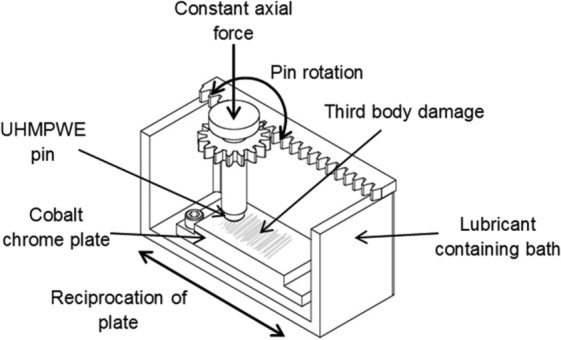
Section view of the lubricant containing bath used in the pin on plate wear test.

A six-station multi-axial pin on plate reciprocating rig^10^ was used to determine the wear of UHMWPE articulating against the CoCr counterfaces damaged with different third-body particles. The damaged CoCr plate was secured in a lubricant containing bath which reciprocated at 1 Hz with a stroke length of ±20 mm. The pin was held in a pin holder which rotated (±20°) via a rack and pinion mechanism and was axially loaded with a constant load of 160 N via a mass carrying cantilever mechanism to give a nominal contact pressure of 8.1 MPa (Figure 3); wear simulation was carried out perpendicular to the direction of the damage simulation.

Wear simulation was performed at room temperature and lubricated with 25% (v/v) bovine serum supplemented with 0.03% sodium azide to retard bacterial growth to give a final protein concentration of approximately 15 g/L. Throughout the test, the level of the lubricant was maintained above the bearing surfaces.

The wear of the UHMWPE pins was determined by their loss in mass by gravimetric analysis. Prior to weighing, the pins were cleaned in 70% propan-2-ol in an ultrasonic bath and allowed to stabilise for 48 h in a temperature and humidity controlled environment. Measurements were taken before the wear test to set a datum, after 115,000 cycles which equates to 6 weeks in vivo based on 1 million cycles (MC) per year^40^ and the duration the BVFs remain in the body before resorption and after 500,000 cycles equivalent to 6 months in vivo for the average patient with a moderate activity level. However, there is a wide variation in the activity levels between patients, and as the demands and demographics of patients change, it is likely that younger, more active patients^41^ will take a number of steps approaching 2 million.^42^ Unloaded soak controls were maintained in the same lubricant as used for testing. Gravimetric measurements were taken using an AT21 digital microbalance (Mettler Toledo Inc, OH, USA) with a readability of 1 µg and a reproducibility of 2 µg. Each pin was weighed until five consecutive measurements were achieved in a range of ±5 µg. The weight loss of the pins was converted to a volume loss (*V*) using the soak controls to compensate for moisture uptake and a density of 0.934 mg/mm^3^ for GUR 1020 UHMWPE.^43^ The wear factor (*k*) was calculated using the sliding distance for the test (*X*) and the applied load (*P*) as shown in equation (1). ^44^

(1) $$k=\frac{V}{PX}$$

The wear of UHMWPE pins against plates damaged with third-body particles was compared to the wear against the highly polished (*R_a_* < 0.01 µm) negative control CoCr plates and positive control plates scratched with a diamond stylus.

Following wear simulation, surface topography measurements were repeated and the mean values with 95% confidence limits calculated. Statistical analysis was carried out using one-way analysis of variance (ANOVA) with Minitab 17^45^ followed by a Tukey’s post hoc test. Data were considered significant for *p* < 0.05.

The data associated with this article are openly available from the University of Leeds Data Repository.^46^

## Results

### Phase 1: third-body damage simulation

**Figure 4.**
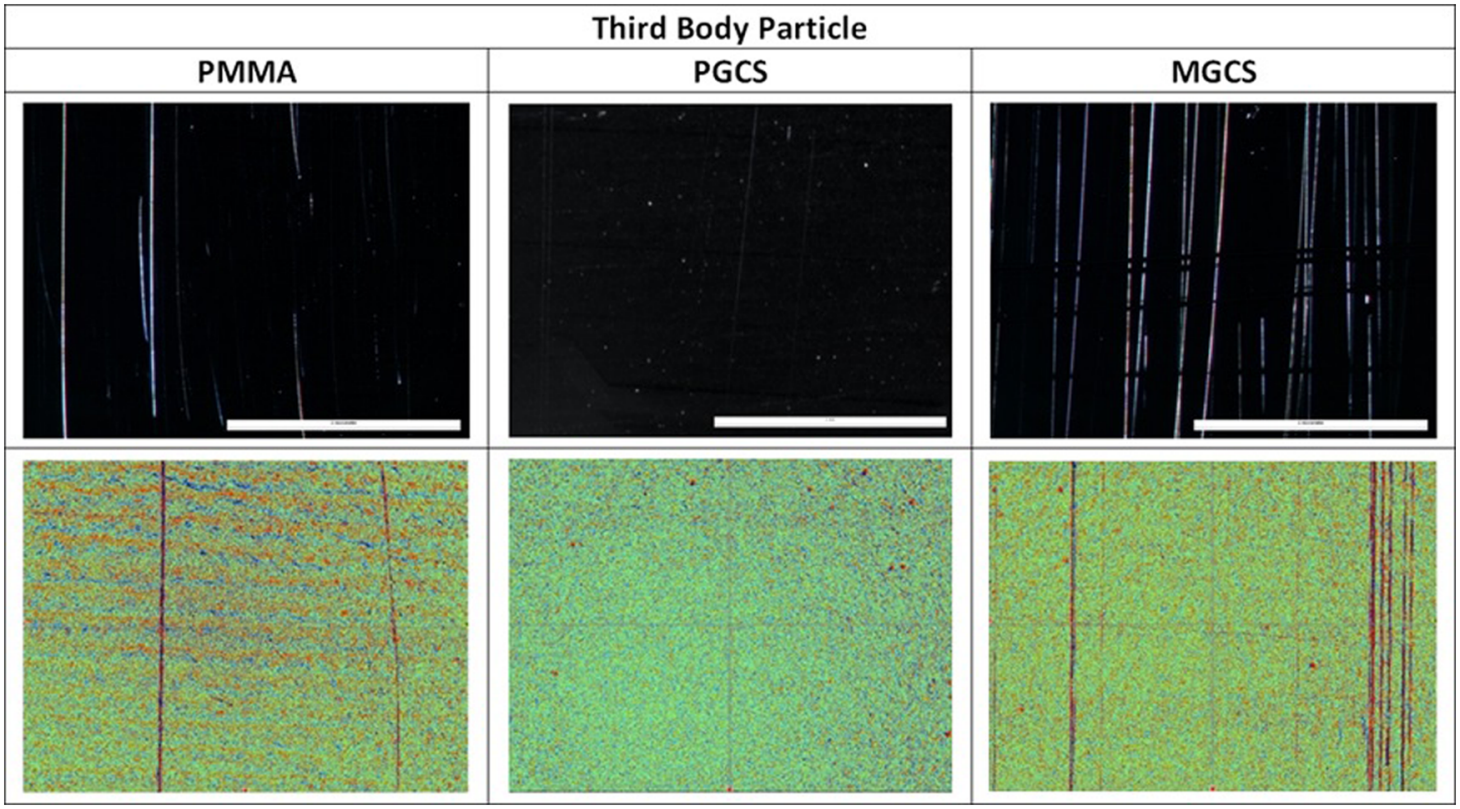
Images of the surface of the cobalt chrome plates at the conclusion of the study. Top: stereomicroscopy images (Nikon SMZ800 stereomicroscope) 63× magnification, scale bar represents 1 mm. Bottom: white light interferometry measurements (Bruker NPFlex) 10× magnification with 2.5× multiplier, analysed with a robust Gaussian Filter, long wave cut-off of 0.04 mm and cylinder and tilt form removal.

Following damage simulation with PMMA and MGCS, scratches were evident on the surface of the cobalt chrome counterfaces as shown in the stereomicroscope images and white light interferometry measurements in Figure 4; no scratches were observed on the plates damaged with PGCS. The number of scratches and their mean lip height were analysed from the white light interferometry measurements, as shown in Table 3. There were no measurable scratches on the surface of the plates damaged with PGCS, the plates damaged with MGCS had 1.62 scratches per mm which was significantly (*p* < 0.05) higher than the number of scratches caused by PMMA. The scratches caused by MGCS had a mean lip height (0.04 ± 0.02 µm) which was similar to those caused by PMMA (0.03 ± 0.05 µm).

**Table 3.** Analysis of scratches on the cobalt chrome plates following third-body damage simulation using the Bruker NPFlex White Light Interferometer (mean ± 95% CL) analysed with a robust Gaussian Filter, long wave cut-off of 0.04 mm and cylinder and tilt form removal.

Scratching material	Number of scratches per mm	Mean lip height (mm)	Mean valley depth (mm)
PMMA	0.185 ± 0.208	0.028 ± 0.051	0.017 ± 0.031
PGCS	0	0	0
MGCS	1.615 ± 1.006	0.036 ± 0.019	0.016 ± 0.007

PGCS: pharmaceutical grade of calcium sulfate; MGCS: medical grade calcium sulfate; PMMA: polymethyl methacrylate.

Prior to damage simulation, the mean surface roughness (*R_a_*) of the plates was <0.01 µm; following third-body damage simulation, the surface topography of the plates was measured using a contacting Form Talysurf in terms of *R_a_, R_p_*, and *R_v_* as shown in Table 4. The mean surface roughness (*R_a_*) of all the plates damaged with third-body particles was similar (*p* > 0.05), and there was no significant difference in the *R_v_* or *R_p_* of the plates following third-body damage. The roughness data shown in Table 4 are filtered as per the ISO standard^47^ to remove the background waviness, but there was a concern that in this case, the filtering led to an underestimation of the surface topography. The primary unfiltered data for the plates damaged with third-body particles are also shown in Table 4 and follow a similar trend to the analysis of the scratches with the white light interferometer, but there was no significant difference in *P_p_* between the plates damaged with different third-body particles.

**Table 4.** Mean (±95% CL) surface roughness (*R_a_* values) of cobalt chrome plates perpendicular to damage following the scratching protocol measured using a contacting Form Talysurf (form removal and a Gaussian filter with 0.8-mm upper cut-off was applied to filter the data). *p* values show mean primary analysis (±95% CL) following damage simulation with least squares line form removal but no filtering.

Scratching material	*R_a_* (µm)	*R_p_* (µm)	*R_v_* (µm)	*P_a_* (µm)	*P_p_* (µm)	*P_v_* (µm)
Negative control	0.005 ± 0.004	0.022 ± 0.018	0.016 ± 0.011			
Positive control	0.219 ± 0.010	1.360 ± 0.146	1.671 ± 0.097	0.243 ± 0.040	2.487 ± 0.244	3.169± 0.199
PMMA	0.006 ± 0.003	0.027 ± 0.022	0.016 ± 0.005	0.075 ± 0.058	0.150± 0.100	0.227 ± 0.159
PGCS	0.004 ± 0.001	0.023 ± 0.015	0.011 ± 0.003	0.057 ± 0.054	0.142 ± 0.095	0.178 ± 0.183
MGCS	0.006 ± 0.005	0.035 ± 0.029	0.018 ± 0.012	0.081 ± 0.089	0.213 ± 0.117	0.282 ± 0.315

PGCS: pharmaceutical grade calcium sulfate; MGCS: medical grade calcium sulfate; PMMA: polymethyl methacrylate.

### Phase 2: experimental wear simulation

**Figure 5.**
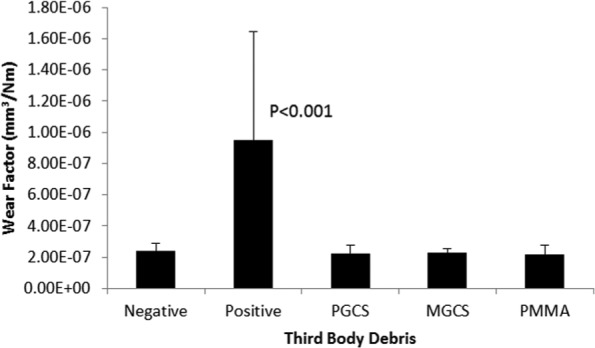
Mean wear factor (±95% CL) of UHMWPE pins after 500,000 cycles of wear simulation. PGCS: pharmaceutical grade calcium sulfate; MGCS: medical grade calcium sulfate; PMMA: polymethyl methacrylate.

Following damage simulation, the wear of UHMWPE articulating against the damaged plates was determined in a six-station pin on plate reciprocating rig perpendicular to the direction of damage simulation. At the conclusion of the wear simulation, there were light scratches on the surface of the plates in the principal direction of sliding and a polished appearance on the articulating surface of the UHMWPE pins where the machining marks had been removed. Figure 5 shows the mean wear factor of the UHMWPE pins articulating against the control plates and those damaged with different third-body particles after 500,000 cycles of wear testing. Statistical analysis by one-way ANOVA showed a significant (*p* < 0.001) difference in the wear of the UHMWPE pins against the different counterfaces although post hoc analysis showed the wear to be similar against the plates damaged with third-body particles and the negative controls and significantly higher against the positive control plates.

## Discussion

The approach used in this study was based on a previous methodology described by Minakawa,^24^ which separated damage simulation and wear testing into two phases in a simple geometry pin on plate configuration. Pin on plate tests cannot replicate all the tribological variations found in a knee replacement but allow a single tribological variable, in this case the influence of surface damage on wear of UHMWPE to be isolated.^6^ In the first phase, damage simulation was carried out using different clinically relevant third-body particles to generate damage more representative of that seen in vivo.^4^ The high conformity of the flat on flat surfaces reduced the potential for the particles to migrate away from the contact site.^18^ In the second phase, wear tests were carried out against the damaged surfaces. It is accepted practice to create surface damage and then to carry out wear testing,^3,5,10^ and by adopting this approach, both the surface topography of the cobalt chrome counterfaces and the wear of UHMWPE pins against the damaged surfaces were assessed.

### Phase 1: third-body damage simulation

There were no measurable scratches on the surface of the cobalt chrome plates damaged with PGCS, the damage created was below the resolution of the objective lens used (vertical resolution <0.15 nm, lateral resolution 0.9 µm);^39^ MGCS caused scratches of the highest density and lip height of the third-body particles studied. The relative differences in the MGCS and PGCS materials have been discussed in literature.^48^ MGCS is manufactured from the purification of naturally sourced gypsum with a typical purity in excess of 98% due to impurities such as calcium carbonate, magnesium carbonate, and “aggregate.”^37^ PGCS is manufactured via a completely synthetic route and as such has a uniform crystalline structure which does not contain the potential for these impurities.^34^ As the only difference between these two materials is the level of impurities as a result of the routes of manufacture, it is postulated that the presence of impurities in the calcium sulfate have an influence on the damage to the cobalt chrome surfaces. Following damage simulation with PMMA bone cement, scratches were visible on the surface of the plates. Several previous studies have investigated third-body damage with particles of PMMA bone cement of varying composition and have shown that during polymerization, the radiopacifiers in the cement agglomerate giving large, abrasive clusters which can damage metal counterfaces. The most commonly used radiopacifier particles in bone cement are zirconium dioxide and barium sulfate, and studies have shown zirconium dioxide to have a more detrimental effect on metallic counterfaces.^8,18,21,49^ The GMV bone cement used in this study contained barium sulfate; however, the magnitude of the surface damage caused by PMMA was lower than that caused by MGCS. A particle size range of 500–1000 µm diameter was used for PMMA bone cement which is consistent with previous experimental wear simulation studies,^20^ and observations of the size of bone cement particles present in the joint following lavage have shown the presence of particles up to 1 mm diameter.^50^ While smaller diameter cement particles (<100 µm) are frequently identified embedded in the polyethylene of retrieved knee replacements and surrounding tissue, it is particles of a larger diameter (∼300 µm diameter) which are considered to cause more severe damage to the surfaces.^51^ The size of the indentations observed in retrieved UHMWPE tibial components of total knee replacements thought to be caused by particles of bone cement suggests that large cement particles may also become entrapped between the articulating surfaces, which break up during the loading and motion of the implant^52^ giving rise to smaller embedded PMMA particles in the UHMWPE.^7,53,54^

It was noted that the different third-body materials behaved differently when loaded between two surfaces. When trapped between the pin and plate, the brittle calcium sulfate BVFs became a fine powder. The PMMA, however, maintained its particulate shape when trapped between an UHMWPE pin and a cobalt chrome plate and the particles were embedded into the pin. Particles of PMMA bone cement have been observed in UHMWPE acetabular cups of retrieved hip replacements^55^ and the tibial components of retrieved total knee replacements.^7,56,57^ The resorbable nature of the BVFs^58^ means that if they were to become trapped between the articulating surfaces of an implant, then over time, they would degrade so the duration the particles would be present in the joint would be relatively short, limiting damage to components. The inert and non-resorbable nature of trapped PMMA particles, however, means that while the magnitude of the damage they caused to the cobalt chrome counterfaces in this study was lower than that caused by MGCS, there is the potential for the embedded PMMA particles to cause continued roughening of the femoral component. As the roughness of the metal counterface has a strong influence on the wear rate of the UHMWPE component, increased abrasion of the UHMWPE will increase the generation of wear debris increasing the potential for osteolysis^59^ and aseptic loosening which will ultimately reduce the life span of the implant.

### Phase 2: experimental wear simulation

Against smooth negative control cobalt chrome plates, the wear of the UHMWPE pins was similar to studies carried out under similar kinematic conditions.^60^ Previous studies have shown damage to the metal counterface resulting in an increased *R_a_* to accelerate wear;^61^ however, a non-linear relationship between *R_a_, R_p_*, or lip height and wear has been exhibited, so that below a critical lip height, wear rate is similar to negative controls; Minakawa et al.^6^ described an exponential relationship between *R_p_* and wear factor showing when the *R_p_* was <0.5 µm, the wear was similar to highly polished controls, but an *R_p_* > 0.5 µm caused a dramatic increase in wear. A non-linear relationship between lip height and wear of non-crosslinked polyethylene was further demonstrated by Galvin et al.^10^ It was hypothesised that the third-body materials that caused surface damage with the highest lip heights would have the strongest influence on the rate of wear of UHMWPE. However, in this study, the damage caused by the third-body particles did not have lip heights of sufficient magnitude to increase the wear of UHMWPE above that of the negative controls. The wear was only significantly increased against positive control plates with a 2 µm lip height.

## Conclusion

Third-body particles originating from PMMA bone cement and calcium sulfate BVFs can damage highly polished cobalt chrome counterfaces, and BVFs of similar composition can cause varying magnitudes of surface damage. Experimental wear simulation using a six-station pin on plate reciprocating rig against the damaged surfaces showed a similar rate of wear for UHMWPE pins articulating against negative (highly polished) plates and plates damaged with third-body particles and the wear rate was only increased in the positive control plates scratched with a diamond stylus (2 µm lip height). This study suggests that if calcium sulfate BVFs are used close to the articulating surfaces of total joint replacements, the purity of the calcium sulfate used may influence the magnitude of the surface damage. However, long-term wear tests of arthroplasty components should be carried out to determine whether the surface damage to cobalt chrome counterfaces caused by calcium sulfate BVFs influences UHMWPE wear volumes and ultimately the life span of total joint replacements.
